# Potentiometric Solid-Contact Ion-Selective Electrode for Determination of Thiocyanate in Human Saliva

**DOI:** 10.3390/s20102817

**Published:** 2020-05-15

**Authors:** Marcin Urbanowicz, Kamila Sadowska, Dorota G. Pijanowska, Radosław Pomećko, Maria Bocheńska

**Affiliations:** 1 Nalecz Institute of Biocybernetics and Biomedical Engineering Polish Academy of Sciences, Ks. Trojdena 4, 02-109 Warsaw, Poland; ksadowska@ibib.waw.pl (K.S.); dpijanowska@ibib.waw.pl (D.G.P.); 2 Department of Chemistry and Technology of Functional Materials, Chemical Faculty, Gdańsk University of Technology, Narutowicza 11/12, 80-233 Gdańsk, Poland; radoslaw.pomecko@pg.gda.pl (R.P.); maria.bochenska@pg.gda.pl (M.B.)

**Keywords:** thiocyanate ion-selective electrodes, potentiometry, thiocyanate in human saliva, ionograms, calix[4]arene

## Abstract

A new solid-contact potentiometric ion-selective electrode for the determination of SCN^−^ (SCN-ISE) has been described. Synthesized phosphonium derivative of calix[4]arene was used as a charged ionophore. The research included selection of the ion-selective membrane composition, determination of the ISEs metrological parameters and SCN-ISE application for thiocyanate determination in human saliva. Preparation of the ISEs included selection of a plasticizer for the ion-selective membrane composition and type of the electrode material. The study was carried out using ISE with liquid internal electrolyte (LE-ISE) and solid-contact electrodes made of glassy carbon (GC-ISE) and gold rods (Au-ISE). The best parameters were found for GC sensors for which the ion-selective membrane contained chloroparaffin as a plasticizer (S = 59.9 mV/dec, LOD = 1.6 × 10^−6^ M). The study of potentiometric selectivity coefficients has shown that the thiocyanate-selective sensor could be applied in biomedical research for determination of SCN^−^ concentration in human saliva. The accuracy of the SCN^−^ determination was verified by testing 59 samples of volunteers’ saliva by potentiometric sensors and UV-Vis spectrophotometry as a reference technique. Moreover, SCN^−^ concentrations in the smokers’ and non-smokers’ saliva were compared. In order to investigate the influence of various factors (sex, health status, taken medications) on the thiocyanate level in the saliva, more extensive studies on a group of 100 volunteers were carried out. Additionally, for a group of 18 volunteers, individual profiles of SCN^−^ concentration in saliva measured on a daily basis for over a month were collected.

## 1. Introduction

Human saliva is one of the body fluids, produced by the salivary glands. The whole saliva is a complex fluid consisting of saliva—an exocrine secretion, gingival fluid and serous exudate [1]. The main role of saliva is to maintain oral homeostasis and to facilitate the initial digestion and swallowing of food [2]. The components of saliva are water, enzymes, proteins and inorganic ions, bacteria, nasopharynx and epithelium [1]. There are three main pairs of salivary glands: parotid, sublingual and submandibular, which produce 95% of saliva volume. The remaining 5% is produced by the tongue glands and other oral glands. In total, healthy salivary glands produce 1–1.5 L of saliva per day [3]. Saliva production is controlled by two mechanisms: blood filtrate into the glands cavity and the proteins’ and proteoglycans’ secretion by secretory part cells [4]. At first, saliva is isotonic, but when it flows through the striated duct, Na^+^ and chloride ions are more reabsorbed comparing to the secretion of K^+^, HCO_3_^−^ and SCN^−^, making the fluid hypotonic. 

The non-traumatic and facile way of saliva sample collection drives the interest of its possible usage in diagnostics [5,6]. In the last two decades, scientists have been engaged in seeking the correlation between the saliva composition and various diseases. The relationship between salivary composition and volume has been demonstrated for several diseases, such as chronic kidney disease (CKD) [7], oral enamel erosion [8], cystic fibrosis (chloride ions concentration) [9], multiple sclerosis (IL-1ß concentration) [10], graft versus host disease (GVHD) manifesting in reduced production of saliva [11], diabetes and HIV. Comparison of the saliva ionic composition from patients suffering from CKD and healthy people proved that the level of K^+^ and HCO_3_^−^ is significantly higher in all examined patients [12]. In addition, patients who had nausea (one of the symptoms of uremia due to CKD) had a higher Na^+^ concentration than those who did not report those complaints. Dryness in oral cavity that occurred in most CKD patients was associated with higher Ca^2+^ level. Clear differences in the concentration of some saliva ions were also shown in the group of patients with malignant parotid gland cancer [13,14]. The content of Ca^2+^ ions was reduced, and the concentration of Mg^2+^ ions was almost twice as high as compared to healthy people. Interestingly, in the plasma of patients tested for cancer, the concentration of Ca^2+^ ions was higher than in healthy people. In a study conducted on a group of smokers, it was proved that the exposure to tobacco smoke increases the concentration of Ca^2+^, NO_3_^-^ and SCN^−^ ions in saliva. 

Thiocyanates take part in the detoxification process of human organism. The thiocyanates are formed in human liver as a result of decomposition of toxic cyanates [15]. The saliva is the body fluid, which contains the highest concentration of SCN^–^ ions, typically from 0.5 to 2 mM [16]. The determination of thiocyanates concentration could be useful in detection of oral cavity inflammation, smokers parametrizing [17] or monitoring the cancer patients during chemotherapy [14,18,19,20,21,22].

The thiocyanates concentration could be determined by photometric [15,23,24,25] or chromatographic methods [26,27,28,29]; in all cases, however, the analytical methods involve complicated measuring systems and/or complicated procedures of samples preparation, because of the complex composition of the saliva matrix [30,31]. As an alternative, the potentiometric method applying ion-selective electrodes (ISEs) has been proposed. The majority of thiocyanate selective ionophores presented in the literature are based on porphyrins [32,33,34,35,36,37] and phthalocyanines [38,39,40]. Some other macrocyclic compounds such as aza-macrocycles [41,42,43], crown ethers [44] or calix[4]arenes [45] were also reported. The second most exploited systems are organic and metalloorganic non-macrocyclic complexes [46,47,48,49,50,51,52,53,54,55,56,57,58,59,60,61,62]. All presented ionophores are characterized by good selectivity over chlorides, but the low selectivity over two other ions present at high levels in human saliva, namely HCO_3_^−^ and H_2_PO_4_^−^. This hinders their application in medical analytics. The comparison of metrological parameters of ISE with different ionophores and electrodes construction reported in the literature is presented in Table S1. 

The main advantage of potentiometry over the other methods is the ability to perform measurements directly in the sample, even an opaque one. The development of technology, as well as application of modern sensor materials, has led to the situation in which ISEs have become one of the basic tools used in diagnostic laboratories [63]. Currently, medical analyzers are equipped with modules for determination of selected ions, such as Na^+^, K^+^, Li^+^, Ca^2+^, Mg^2+^, Cl^−^ and H^+^ ions, mainly in body fluids such as blood and urine [64]. Since our priority is application of the sensors in bioanalysis and their technology allowing for miniaturization, then our research concerns sensors, being a type of coated wire electrodes (CWEs), and that could be used for measurements in biological samples. The description of the complexity of the problem of such determinations, namely anions in biological samples, can be found in the article by Lo Nostromo [65].

In this paper, characterization and comparison of ISEs with an ion-selective membrane based on tetrakis-(4-diphenylmethylphosphonium-butoxy)-tetrakis-*p*-tert-butylcalix[4]arene tetra-thiocyanate is reported. Described here solid-contact ion-selective electrodes are a type of CWEs, in which an electroactive species is entrapped in a thin polymeric layer deposited directly onto metallic or non-metallic conductor, forming a non-symmetrical potentiometric half-cell (solution|membrane|conductor) [66]. The research was carried out using three different SCN-ISE designs: classic with liquid internal electrolyte (LE-ISE), and solid-contact using glassy carbon (GC-ISE) and gold (Au-ISE) as the base electrode materials. The various compositions of the ion-selective membrane have been studied in terms of the type of plasticizer. The metrological parameters of all types of the developed SCN-ISE were determined. The miniaturized Au-ISEs were used to determine SCN^−^ in volunteers’ saliva samples. The results of the potentiometric and of the UV-Vis analysis were compared. 

## 2. Materials and Methods

### 2.1. Chemicals

**Figure 1:**
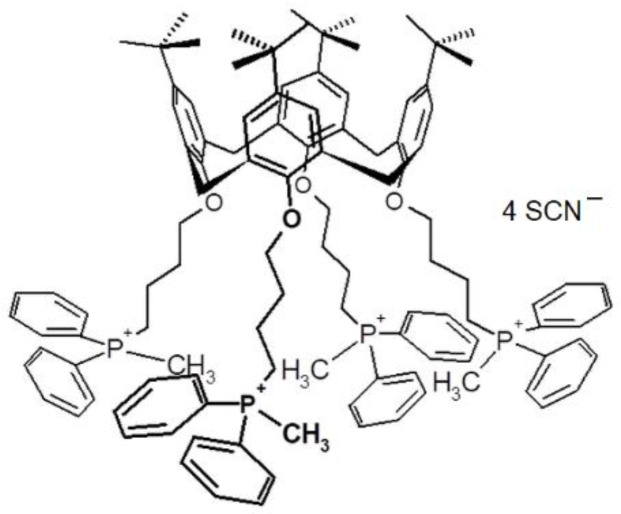
Molecular formula of tetrakis-(4-diphenylmethylphosphonium-butoxy)-tetrakis--p-tert-butylcalix[4]arene tetrathiocyanate.

The ionophore, tetrakis-(4-diphenylmethylphosphonium-butoxy)-tetrakis-p-tert-butylcalix[4]- arene tetrathiocyanate (Figure 1) was synthesized at Faculty of Chemistry of Gdansk University of Technology according to the procedures given elsewhere [67]. PVC (high molecular weight poly(vinyl chloride)), tetrahydrofuran (THF), 2-nitrophenyl octyl ether (*o*-NPOE), *bis*-(butylpentyl)adipate (BBPA) and chloroparaffin were selectophore grade and obtained from Sigma-Aldrich (Merck KGaA, Darmstadt, Germany). The sodium salts: Cl^−^, Br^−^, I^−^, ClO_4_^−^, SCN^−^, NO_3_^−^, SO_4_^2−^, CO_3_^2−^, H_2_PO_4_^−^, acetate, benzoate were of analytical grade (Avantor Perfomance Materials S.A., Gliwice, Poland). All aqueous solutions were prepared with ultra-pure water (18.2 MΩ·cm). 

### 2.2. Equipment

The EMF measurements were carried out at 20 °C, using Precision Electrochemistry EMF Interface, EMF-16 Lawson Labs Inc. A double-junction electrode Orion ROSS Ultra 800500U D/J from Thermo Scientific® (Waltham, MA, USA) was used as a reference electrode. The calibration of electrodes was carried out with the system of automatic biurets TITRONIC® Universal (Schott Instruments, Germany), controlled by PC computer. The UV-Vis measurements were carried out on UV300 UV-Visible Spectrometer Unicam. The Ag/AgCl Phillips electrodes bodies of ISE-561 type (Glasblaserei Moeller AG, Zurich, Switzerland) were applied to build the classical ion-selective electrodes with liquid electrolyte (LE-ISE). A base for the glassy carbon electrodes ISE constituted the bodies produced by Mineral (Warsaw, Poland). The gold electrodes were made from a 500-μm Au wire, which was placed in a poly(methyl methacrylate) casing. The overall diameter of the Au-ISE with case was 2 mm and the GC-ISE 6 mm. Detailed description of the construction is given in our previous reports [68,69,70]. 

### 2.3. Thiocyanate-Solid State Ion Selective Electrodes (SCN-ISE)

The thiocyanate selective membranes were composed of 2.1 wt.% ionophore, 30.7 wt.% PVC, and 67.2 wt.% plasticizer (mass of membrane 200 mg). All the components were dissolved in 1.5 mL of THF. To form an ion-selective membrane, the membrane cocktail was poured into a glass ring of 24 mm in diameter. The solutions were left for slow solvent evaporation (for 24 h), giving the master membrane of a thickness of about 0.1 mm. Several membranes of a 7-mm diameter were cut out from the master membranes and incorporated into Ag/AgCl electrodes bodies of ISE-561 type. The solution of 1 mM KCl was used as an internal electrolyte. The surfaces of GC electrodes (diameter of 2 mm) were prepared by polishing with sandpapers of growing grit from 600 to 4000. The final polishing was performed with Al_2_O_3_ powder (grain size 0.3 µm). The surfaces of GC electrodes were chemically cleaned by sequential washing with solutions: 1 M KOH in MeOH, deionized water, 1 M HNO_3_, and again with deionized water [71]. The 30 µL of previously described membrane solution was poured directly on the prepared GC surface and left for 24 h to evaporate and form a membrane. After that time, to complete Bakker protocol on the determination of unbiased selectivity coefficients which is generally applicable independent of the nature of primary or interfering ions [72], the prepared GC, Au and LE membrane electrodes were left in a 1 mM KCl water solution for the next 24 h. Surfaces of gold electrodes were prepared using an analogous procedure to that for GC electrodes. Due to the much smaller diameter of the Au electrodes (500 µm), the volume of the applied membrane was 3 µL. The studies were repeated several times over the period of four months. The calibration curves were determined by EMF measurement of ion concentration, during gradual dilution of stock solutions from 100 to 0.1 µM. The given low detection limits are taken as the activity of thiocyanates at the point of intersection of the extrapolated linear midrange and final low concentration level segments of the calibration plot [73,74]. The selectivity coefficients were determined by a separate solution method (SSM) [75] and a fixed interference method (FIM) [76,77].

### 2.4. Saliva’s Sampling and Measurement Protocol

Saliva samples were taken from volunteers using a specially prepared kit. The sampling kit contained a string pouch, a 5 mL sterile syringe (Polfa SA Lublin, Lublin, Poland) and a 3 cm^2^ piece of parafilm to secure the syringe outlet. A sticker with an identification code was attached to the syringe. Thirty minutes before the sample was taken, the volunteers could not eat and drink, and about 2 mL of saliva was collected from each one. After collection, the samples were centrifuged (6 min, 6000 rpm), and then immediately used for the determination of SCN^−^. To eliminate the effect of the day time on the measurement results, the samples were collected between 10 and 11 a.m. Saliva samples were taken from 100 volunteers. For 18 volunteers, saliva samples were collected each day for over a month. The group of 100 volunteers were students aged 20–25. Before saliva sampling, each volunteer had to respond to the anonymous survey (Supplement). Based on the surveys, it was possible to correlate the levels of measured SCN^−^ with specific aspects of health and lifestyle. The entire study group included 68% men and 32% women. Over half of the respondents reported the feeling of general fatigue. Thirty percent of the respondents reported that they suffer from various types of chronic diseases. The largest group were volunteers with allergic (23%) and pulmonary (20%) diseases. Volunteers taking medications or dietary supplements constituted 69% of the group. The most commonly used medicaments were those from the over-the-counter (OTC) group (32%) and supplements (30%). Contraceptives were taken by 17% of the women. Daily oral hygiene was declared by 95% of respondents, and the use of oral care liquids 27%. The daily volume of fluids consumed fluctuated between 2 and 2.5 L, and the most frequently consumed fluid was water. UV-Vis spectrophotometry was used as a reference method for SCN^−^ determination. A part of saliva supernatant was prepared according to the procedures reported in [15] and then analyzed by the spectrophotometric method, described therein, in particular with measuring of the absorbance of the solution at 447 nm against the Fe(NO_3_)_3_ stock solution.

## 3. Results

### 3.1. SCN-ISE Parameters

**Figure 2:**
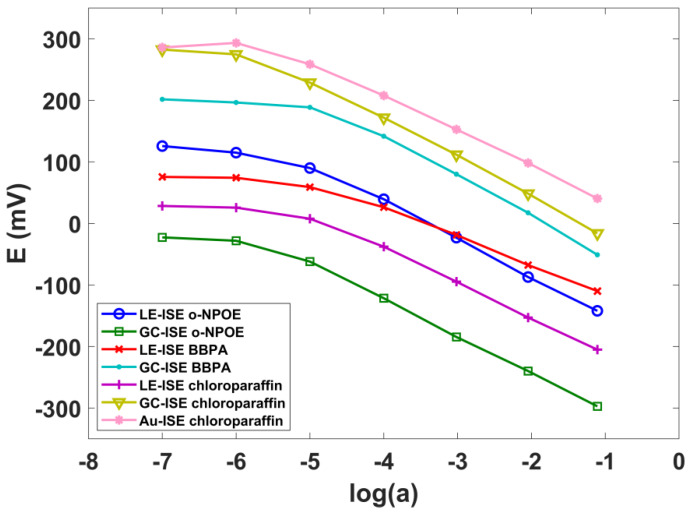
The LE-ISE, GC-ISE and Au-ISE characteristics for full measurement range of SCN^−^ ion activity.

In order to obtain the best composition of the ISE membrane for thiocyanate detection, the influence of three plasticizers, *o*-NPOE, BBPA and chloroparaffin, was investigated. The type of plasticizer may affect the metrological parameters of ISEs. A change of the plasticizer polarity has an influence on the selectivity and sensitivity of the ion-selective membrane as it affects the dielectric constant of the membrane and ion-ionophore complex binding constant [78]. For each plasticizer, the LE-ISE and GC-ISE electrodes were prepared and their characteristics were determined (Figure 2). Among the tested electrodes, those containing BBPA in the membrane composition were characterized by the worst parameters. In the case of LE-ISE (BBPA), the sensitivity was −47.7 ± 3.8 mV/dec while detection limit expressed as a logarithm log(*a_SCN_*) was equal to −4.67. In the case of GC-ISE (BBPA) electrodes, the parameters were −61.9 ± 3.2 mV/dec and −4.82, respectively. As can be seen in Table 1, the best sensor parameters among studied electrodes, revealed those with chloroparaffin as a plasticizer. 

In the case of LE-ISE, the obtained detection limit expressed as log(a) was equal −5.2 with sensitivity −55.5 ± 2.1 mV/dec, whereas the parameters of GC-ISE electrode were even better and equaled to −5.8 and 59.9 ± 0.3 mV/dec, respectively. Au-ISE sensitivity reached −53.3 ± 2.1 mV/dec and was the least favorable as compared to other constructions. 

The differences in SCN-ISE metrological parameters based on charged calix[4]arene derivatives result from ISEs construction including LE-ISE and coated wire electrodes (CWE) type Au and GC-ISE, where two kinds of interfaces are present: symmetrical—solution|membrane|internal solution and asymmetrical—solution|membrane|conductor, respectively. Therefore, the main problem related to the CWE type of sensors is to establish the thermodynamically well-defined, reversible potential, that depends on charge transfer mechanism at the conductor–membrane interface [66]. The charge transfer is determined by the properties of the conducting material. Then, different types of CWE constructions (Au and GC) with the same membrane composition showed some differences in their metrological parameters. In addition, plasticizers have a significant influence on the ISEs potentiometric response. This phenomenon is associated with a change in the electrical properties and lipophilicity of the ion-selective membrane that affect ISEs’ parameters.

The lifetime of the electrodes was determined by recording their potentials and by plotting the calibration curves for each week. In the case of ISE electrodes plasticized with *o*-NPOE or chloroparaffin, no significant changes in the sensitivity were observed for three months. After that time, however, a gradual decrease of electrodes sensitivity was observed. 

The determined selectivity (FIM) of LE-ISE containing chloroparaffin in the membrane follows the series: ClO_4_^−^ > SCN^−^ ≥ I^−^ > NO_3_^−^ > Benzoate^−^ > Br^−^> Acetate^−^ > HCO_3_^−^ > Cl^−^ > H_2_PO_4_^−^, while the selectivity pattern of GC-ISE with the same plasticizer was: ClO_4_^−^ > SCN^−^ ≥ I^−^ > NO_3_^−^ > Acetate^−^ = Br^−^ > Benzoate^−^ = HCO_3_^−^ = Cl^−^ = H_2_PO_4_^−^. The selectivity coefficients of the SCN-ISE were compared in Table 2. 

The selectivity coefficients were determined for all tested electrodes. The work parameters predestine the LE-ISE, GC-ISE and Au-ISE containing chloroparaffin as the electrodes for analytical applications. Considering the possibility of using SCN-ISE in biomedical applications, an analysis of the required selectivity coefficients was performed. The analysis included the physiological concentrations of individual anions in human saliva. The following equation was used for the calculations [77]:

(1) log(KSCN/jpot req.)=aSCNajzSCNzj·(pSCN/j100%)zSCNzj

where $\log(K_{SCN/j}^{pot\;req.})$ – required potentiometric selectivity coefficient, *a_SCN_* - ion activity for SCN^−^ (value adopted for calculations SCN^−^ = 1 mM), *a_j_* - activity of interfering ion, *z_SCN_,_j_* - charge of SCN^−^ and interfering ion, *p_SCN_,_j_* - relative error (expressed in %) for potentiometric measurements assumed here to be ±2.5%. Table 3 compares the values of the selectivity coefficients (Au-ISE) for individual interfering ions with the calculated values of required selectivity coefficients.

The required selectivity coefficient depends on the ratio of main and interfering ion concentration. Obviously, the higher concentration of target ions results in the interfering ions having a smaller effect on the electrode response. In terms of practical usage of ISEs in clinical analysis, the ratio of concentrations of main ion to interfering ion present in the biological sample is very important, as shown in the Introduction. Assuming the statement given above, the most interfering ion is Cl^−^ which is present in saliva at 20–60 mM level (representative 30 mM) [79]. The second important interferent is HCO_3_^−^ whose concentration in human saliva is also about 30 mM [80]. Taking it into account, the required selectivity coefficient for those ions should be −3.1 or more negative. The selectivity coefficient for Cl^−^ and HCO_3_^−^ ions determined for Au-ISE was −3.9 and −3.4, respectively. Despite the unfavorable selectivity for ClO_4_^−^ and I^−^, the presented electrodes can be applied in the potentiometric bio-measurements, because the concentrations of mentioned interfering anions in saliva are at very low levels, in particular at 1.3 × 10^−^^8^ M and 1.4 × 10^−^^9^ M, respectively [82,83]. These differences in the ion concentrations, at least three orders of magnitude, are enough to allow the proper determination of thiocyanates. Therefore, both types of the developed solid-contact ISEs can be successfully used for direct determination of thiocyanates in saliva samples. 

It should be underlined here, that majority of papers states the possibility of saliva samples analysis but surprisingly only some of them presents selectivity coefficients for bicarbonates [43,48,52,56,61], which is an important anionic component of saliva. Moreover, in the vast majority of the reports, given values of the selectivity coefficients are determined by SSM method, despite other recommendations given by IUPAC [85]. 

### 3.2. SCN^−^ Determination in Human Saliva

**Figure 3:**
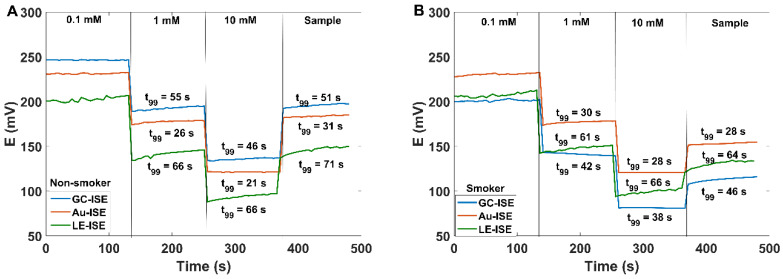
Potentiometric responses of GC-ISE (blue line), Au-ISE (red line) and LE-ISE (green line) to the rapid change of SCN^−^ ion concentration from 0.1 to 1 mM, and next to 10 mM, and then exposure to saliva sample (**A**) for a non-smoking person and (**B**) for a smoker. Mean value of SCN^−^ concentration determined by all type sensors is equal to (**A**) 0.8 ± 0.1 mM and (**B**) 2.8 ± 0.1 mM.

Both metrological parameters of Au-ISEs and GC-ISE, such as lower detection limit and sensitivity, together with the selectivity coefficients allow reliable measurement of SCN^−^ concentration in the presence of other anions present in human saliva (Table 3). The SCN^−^ concentration in the saliva of healthy, non-smoking person is in the range of 0.5−2 mM (representative 1 mM), while it can be several times higher, e.g., for smokers. Therefore, before studies the sensors were calibrated in the range of 0.1−10 mM. Figure 3 shows the GC-ISE Au-ISE and LE-ISE responses to SCN^−^ concentration changes from 0.1 to 10 mM. All types of sensors were used to analyze the saliva sample collected from the same volunteer. Figure 3 clearly shows the consistency of the obtained results for both sensors. Moreover, the level of thiocyanates determined by the reference colorimetric method was equal to 0.82 mM, which is absolutely in agreement with the data obtained by potentiometric method (GC-ISE 0.9 mM; Au-ISE 0.8 mM; LE-ISE 0.8 mM). Response time (t_99_) for GC-ISE, Au-ISE and LE-ISE was 51, 31 and 71 s, respectively.

As mentioned before, one of the sources of thiocyanates in the human body are the cyanides, transformed to SCN^−^ during the detoxification process. The highest amount of cyanides enters the human body with polluted air and tobacco smoke. Therefore, measuring the level of thiocyanates in saliva allows to distinguish smokers from non-smokers. All three sensor constructions (LE, Au and GC) were used to determine the SCN^−^ concentration in the saliva samples donated by smokers and non-smokers. Collected saliva samples were additionally analyzed by the reference UV-Vis method. The results of the experiment are presented in Table 4. As expected, in the smokers’ saliva the SCN^−^ concentration was almost seven times as high as recorded for non-smokers. In addition, the results obtained using three various sensor constructions were consistent with the results of the reference test, which confirms the possibility of using the developed sensors in biomedical measurements, especially in human saliva samples. 

In order to compare the accuracy of the SCN^−^ determination, a series of human saliva samples were analyzed using Au-ISE, along with the reference colorimetric method. Au-ISEs were used as they show comparable parameters as GC-ISEs, but they are more facile in construction, cheaper and allow a smaller amount of the sample to be analyzed. It should be underlined here that, in contrast to other studies, the analyzed saliva samples were neither diluted nor was the pH adjusted. The measurements were performed directly in the saliva sample. Moreover, existing clinical analyzers are mainly based on gold sensors. Therefore, the miniaturized Au-ISEs are convenient for a single measurement, as well as a potential part of the multiplex analysis. Therefore, the more extensive research was carried out using Au-ISEs. 

**Figure 4:**
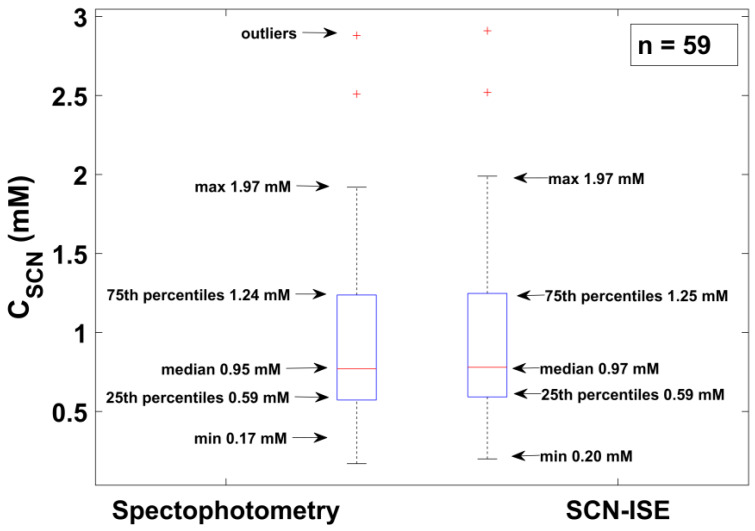
Statistical comparison of the results recorded using the Au-ISE (right box) with the UV-Vis spectrophotometry reference method (left box), (n = 59).

The first analysis was performed on a group of saliva samples collected from 59 volunteers, including four smokers (as they declared in the survey). Figure 4 presents a box-plot comparing the statistical parameters (median, 25 and 75 percentiles, maximum and minimum values) of thiocyanate concentrations determined by the potentiometric method and by the reference UV-Vis analysis. The results obtained by both methods coincided with each other, and the mean and relative median error for SCN^−^ level (n = 59) for the potentiometric method relative to the spectrophotometric method was about 2%. In Figure 4, two outliers can be seen. Two out of four declared smokers manifested increased SCN^−^ content (above 2 mM) in their saliva samples. 

**Figure 5:**
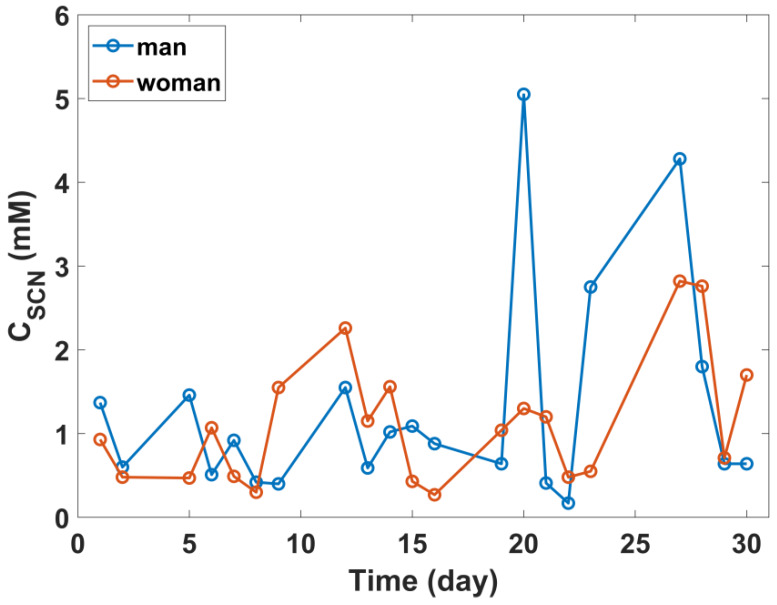
Exemplary ionograms showing fluctuations in SCN^−^ concentration in the saliva of a woman and a man—volunteers taking part in the 30-day study.

In the second study, Au-ISE were used to determine the thiocyanate concentration in 100 saliva samples, each sample donated by one non-smoking volunteer. All volunteers had to respond to the questionnaire. Table S2 (*ESI*) summarizes the collected information and is divided into subgroups of volunteers depending on responses declared in the survey and the mean SCN^−^ concentration along with the standard deviation for individual subgroups. The statistical analysis based on an analysis of variance (ANOVA) did not show statistically significant differences between individual subgroups. In some cases, the subgroup size was too small to be able to perform the correct analysis (e.g., in the case of chronic diseases, taken medications or the last time to brushing teeth). However, taking into account differences in mean values and standard deviations for individual subgroups, it can be concluded that the SCN^−^ concentration is a personal feature and depends primarily on the specific case. In order to gain a detailed insight into the long-term fluctuations of SCN^−^ concentration, a group of 18 (11 women and seven men) volunteers have been examined for 30 days. The daily collected saliva samples (except non-working days) were immediately analyzed in terms of SCN^−^ concentration, using Au-ISEs. Figure 5 shows exemplary ionograms for a man and a woman from the group subjected 30-day study.

The obtained results indicate that both the mean SCN^−^ concentration and fluctuations are the individual features and depend on many factors (including health, physical effort, the environment and the quality of the air). Therefore, the dynamics of these changes vary significantly for the tested group. The *ESI* includes other ionograms, presenting SCN^−^ fluctuation in the saliva of individual volunteers. They confirm the individual nature of these fluctuations, which is independent of, for example, sex. The fluctuation of SCN^−^ for W3 and M3 (Figures S1 and S2) are characterized by the highest dynamics, which may result from, among others, the lifestyle, intensity of effort or diet. Analogous behavior has been previously observed for other ions, Na^+^, K^+^, Cl^−^ [68,69]. In contrast, fluctuations recorded for volunteers W10 and M7 (Figures S1 and S2) are characterized by the lowest dynamics during the month, which can be interpreted as results of volunteers leading a stable and standardized life. The study showed the existence of many intermediate cases, examples of which are volunteers W4, W5, W9, M2, M5, M6 (Figures S1 and S2), for which a single change in SCN^−^ concentration in relation to the monthly mean value was observed. The described changes may be accidental, e.g., related to dehydration, stress as well as a change in the environmental situation; being in the vicinity of smokers or staying longer in the vicinity of a busy street and inhaling air containing a greater content of cyanides, which are later processed into the body to less toxic thiocyanate. 

## 4. Conclusions

Potentiometric sensors in the form of solid-contact ion-selective electrodes for the determination of thiocyanate in human saliva were described. The comparison of metrological parameters for three constructions (LE-ISE, GC-ISE, Au-ISE) showed that the most advantageous parameters were obtained for glassy carbon sensors as the electrode material (S = 59.9 mV/dec, LOD =1.6 × 10^−6^ M). In addition, the phosphonium derivative of calix[4]arene as an ionophore enabled direct SCN^−^ measurement in human saliva samples. The SCN-ISE presented in the work shows more favorable selectivity coefficients in relation to ions occurring at high levels in human saliva (Cl^−^, HCO_3_^−^, H_2_PO_4_^−^). The values of logarithms of the selectivity coefficients confirm that the phosphonium derivative of calix[4]arene can compete with the ionophores based on the porphyrin ring, despite the logarithm of the selectivity coefficients for ClO_4_^−^ has positive value. Despite slightly worse metrological parameters, Au-ISE was used for extensive research on human saliva, mainly due to the shorter response time and comparable parameters with GC-ISE. A reference study has explicitly confirmed that the results obtained with potentiometric sensors are consistent with the results obtained using the UV-Vis reference method. Further research confirmed the significant effect of smoking on the concentration of thiocyanate in human saliva. Despite the salivary analysis of 100 volunteers, no statistically significant differences in SCN^−^ concentration between individual subgroups have been seen. The analysis carried out on a group of 18 volunteers for 30 days confirmed the individual nature of the SCN^−^ ions fluctuation, which may be due to many external factors (passive smoking, diet, chronic diseases, taken medications). The usefulness of the constructed sensors for determining SCN^−^ concentration in human saliva samples was finally confirmed.

## Figures and Tables

**Table 1:** The comparison of SCN-ISEs parameters for three types of the electrodes: LE, GC and Au.

ISE Type	S (mV/dec)	Low Limit of Detection (log*a_SCN^−^_*)	Lifetime (Month)
LE-ISE_BBPA_	−47.4 ± 3.8	−4.67	< 1
GC-ISE _BBPA_	−61.9 ± 3.2	−4.82	< 1
LE-ISE _o-NPOE_	−60.6 ± 1.7	−5.20	3
GC-ISE _o-NPOE_	−60.4 ± 1.2	−5.20	3
LE-ISE _Chloroparaffin_	−55.5 ± 2.1	−5.20	3
GC-ISE _Chloroparaffin_	−59.9 ± 0.3	−5.80	3
Au-ISE _Chloroparaffin_	−53.3 ± 2.1	−5.50	3

**Table 2:** Potentiometric selectivity coefficients for SCN-ISE with chloroparaffin as a plasticizer determined by SSM and FIM methods.

ISE Type	Separate Solution Method $\log K_{SCN/j}^{pot}$ (SD ± 0.1)									
	Cl^−^	H_2_PO_4_^−^	HCO_3_^−^	Ac^−^	Bz^−^	NO_3_^−^	Br^−^	I^−^	SCN^−^	ClO_4_^−^
LE-ISE	−3.2	−4.5	−3.4	−3.1	−2.0	−1.6	−2.0	−0.2	0.0	0.7
GC-ISE	−4.0	−4.0	−3.9	−2.1	−3.8	−1.6	−2.5	−0.3	0.0	0.7
Au-ISE	−3.8	−4.2	−3.0	−2.4	−3.6	−2.0	−2.6	0.2	0.0	0.8
	Fix Interference Method $\log K_{SCN/j}^{pot}$ (SD ± 0.1)									
LE-ISE	−3.9	−4.6	−3.4	−2.7	−2.0	−1.8	−2.1	−0.4	0.0	0.6
GC-ISE	−3.9	−4.2	−3.9	−2.2	−3.9	−1.8	−2.2	−0.5	0.0	0.5
Au-ISE	−3.8	−4.4	−3.6	−2.3	−3.6	−1.8	−2.2	−0.4	0.0	0.6

**Table 3:** Comparison of required potentiometric selectivity coefficients and obtained for the developed solid-contact SCN-ISE, and ranges of selected anions concentration in human saliva.

Interfering Ion	Selectivity Coefficient for Au-ISE $\log(K_{SCN/j}^{pot})$	Required Selectivity Coefficient $\log(K_{SCN/j}^{pot\;req.})$	Representative Interfering Ion Concentration in Human Saliva (mM)
Cl^−^	−3.9	−3.1	30 [79]
HCO_3_^−^	−3.6	−3.1	30 [80]
H_2_PO_4_^−^	−4.4	−2.6	10 [81]
AcO^−^	−2.4	−1	0.24 [31]
Bz^−^	−3.2	1	2.5 × 10^−3^ [82]
NO_3_^−^	−1.8	−1.3	0.76 [31]
Br^−^	−2.2	2.8	4 × 10^−5^ [82]
I^−^	−0.4	4.2	1.4 × 10^−6^ [83]
ClO_4_^−^	0.6	3.3	1.3 × 10^−5^ [84]

**Table 4:** Comparison of SCN^−^ concentrations for non-smokers’ and smokers’ saliva.

Sample	Concentration of SCN^−^ (mM)			
	LE-ISE	GC-ISE	Au-ISE	UV-Vis
I (non-smoker)	0.85 ± 0.14	0.87 ± 0.09	0.79 ± 0.06	0.82 ± 0.01
II (smoker)	5.31 ± 0.06	5.38 ± 0.05	5.49 ± 0.06	5.50 ± 0.01

## Supplementary Materials

The following are available online at https://www.mdpi.com/1424-8220/20/10/2817/s1, Table S1. Comparison of the metrological parameters of different SCN-ISE reported in the literature (listed in chronological order). Reference numbering is in accordance with Reference list in the main manuscript. Table S2: Comparison of the mean concentrations of SCN^−^ in the relation of responses given in the survey. Figure S1: Monthly ionograms showing fluctuations of SCN^−^ concentration for selected women. Figure S2. Monthly ionograms showing fluctuations of SCN^−^ concentration for selected men.
