# Supplementary material for: Potentiometric Solid-Contact Ion-Selective Electrode for Determination of Thiocyanate in Human Saliva
Source: Sensors (Basel). 2020 May 15;20(10):2817. doi: 10.3390/s20102817 (PMC7288078; doi:10.3390/s20102817)
Supplement: Supplementary file 1 [file sensors-20-02817-s001.pdf]

sensors

Potentiometric Solid-Contact Ion-Selective Electrode for Determination of Thiocyanate in Human Saliva

Marcin Urbanowicz [1,*], Kamila Sadowska [1], Dorota G. Pijanowska [1], Radosław Pomećko [2], Maria Bocheńska [2]

[1]Nalecz Institute of Biocybernetics and Biomedical Engineering Polish Academy of Sciences, Ks. Trojdena 4, 02-109 Warsaw, Poland
[2]Department of Chemistry and Technology of Functional Materials, Chemical Faculty, Gdańsk University of Technology, Narutowicza 11/12, 80-233, Gdańsk, Poland
*Correspondence: e-mail: murbanowicz@ibib.waw.pl

Table S1. Comparison of the metrological parameters of different SCN-ISE reported in the literature (listed in chronological order) with indicated compounds group to whom the ionophores belongs. Reference numbering is in accordance with Reference list in the main manuscript. Group of compounds: **(I-1)** porphyrins, **(I-2)** phthalocyanines, **(I-3)** aza-macrocycles, **(I-4)** crow ethers, **(I-5)** calix[4]arenes, **(I-6)** organic and metalloorganic non-macrocyclic complexes. The selectivity coefficients for ions present in the highest concentration in saliva samples, that is Cl^- and HCO_3^- are marked with red.

Ionophore	Membrane/Electrodes System	Method: Selectivity Coefficients	Slope LOD Linear range	Analytical Usage	Ref.
nitron − thiocyanate ion−pair complex **(I-6)**	92 Orion electrode barrel with an Orion 92−06−04 microporous membrane<br><br>Ag/AgCl ǀ10^{-2} M NaSCN, 10^{-2} M NaCl ǀ10^{-3} M nitron−SCN in nitrobenzene ǀǀ porous membrane ǀǀ SCN^- in the test solution ǀAg/AgCl reference electrode	**FIM:** F^- −2.9; Cl^- −2.9; Br^- −2,7; I^- −1.7; ClO_4^- +0.3; IO_4^- +0.5; S^{2-} −0.6; SO_3^{2-} −3.0; SO_4^{2-} −3.0; $S_2O_3^{2-}$ −2.9; $S_2O_8^{2-}$ −2.8; CN^- −2.8; CH_3COO^- −3.0 NO_3^- −2.1 NO_2^- −3.0; $H_2PO_4^-$ −2.0; WO_4^{2-} −2.2; MoO_4^{2-} −2.1; $Fe(CN)_6^{3-}$ −3.6; $Fe(CN)_6^{4-}$ −3.6; Oxalate −2.8; Formate −2.0	−55 mV/dec ~ 10^{-5} M 10^{-1} – 10^{-5} M	Titration of Ag^+ and Hg^{2+} ions with 10^{-2} M NaSCN solution.	[47]
α,α,α,α−chloro(5,10,15,20−tetrakis−(8−bromo−l−naphthyl) porphyrinato)manganese(III) **(I-1)**	1% ionophore, 66%, dibutyl sebacate (DBS), 33% PVC	**SSM**: Cl^- −3.5; Br^- −3.3; I^- −1.9; Sal −1.5; IO_4^- −2.9; ClO_4^- −3.1	−59 mV/dec LOD− no data	Electrode tested towards Cl^-, no experiments with SCN^-.	[33]

	Hg/Hg_2Cl_2(s)KCl(sat.)/MES (0.05 mol/L), pH 5.5∣ sample solution ∣ membrane ∣ KCl (0.1 mol/L), AgCl(s)/Ag		$1.6\cdot10^{-2}$ – 10^{-5} M	No real samples.	
5,10,15,20−tetrakis(2,4,6−triphenylphenyl)porphyrinato)manganese(Ill) chloride **(I-1)**	1% ionophore, 66% DBS, 33% PVC<br><br>Hg/Hg_2Cl_2(s); KCl, 4 M/MES buffer/sample solution/ionselective membrane/MES buffer; NaSCN 50 μM, AgCl(*s*)/Ag	**SSM:** Sal^- −1.6; ClO_4^- −2.3; I^- −3.0; CN^- −3.2; Br^- −3.8; Cl^- −4.6; AcO^- −4.2	Near Nernstian (no value given) LOD− no data 10^{-3} – 10^{-6} M	Saliva samples diluted 1:50 with MES buffer.	[34]
μ−Oxotetraphenyl porphyrinatoiron [$(FeTPP)_2O$] **(I-1)**	1% ionophore, 66% DDS, 33% PVC<br>1% ionophore, 66% DBP, 33% PVC<br><br>Hg/Hg_2Cl_2, KCl (sat.) ∣ samples solution ∣∣ membrane ∣∣ 0.1 M KCl Ag/AgCl	**SSM:** I^- −2.17; ClO_4^- −2.34; NO_2^- −2.73; Br^- −2.92; Cl^- −3.04; NO_3^- −3.70;<br><br>**SSM**: I^- −1.30; ClO_4^- −0.6; NO_2^- −2.00; Br^- −2.50; Cl^- −2.90; NO_3^- −2.3;	−53 mV/dec $3.98\cdot10^{-7}$ M 10^{-1} – 10^{-6} M<br><br>−58 mV/dec $6.31\cdot10^{-5}$ M 10^{-1} – 10^{-4} M	Urine samples diluted 1:10 with 0.01 M H_3PO_4−NaOH solution.<br><br>Not specified, which electrode was used.	[35]
chloro[5,10,15,20−tetra(*p*−methoxyphenyl)porphyrinato]manganese(III) **(I-1)**	1% ionophore, 66% didecyl sebacate (DDS), 33 % of PVC<br><br>Hg/Hg_2Cl_2(s)KCl (sat.)∣ sample solution/membrane/KCl (0.1 mol/L), AgCl(s)/Ag	**SSM**: Cl^- −3.2; Br^- −2.8; I^- −2.4; Sal −1.0; ClO_4^- −2.4; NO_3^- −3.3; NO_2^- −2.8;	Slope: no data $2\cdot10^{-5}$ M 10^{-1} – 10^{-4} M	No real samples.	[36]
cobalt phthalocyanine **(I-2)**	10 % ionophore, 30% BEHP, 60% PVC;	**SSM**: SO_4^{2-} −4.08; $H_2PO_4^-$ −3.07; CO_3^{2-} −2.98; CH_3COO^- −2.03; Oxalate	−59.1 mV/dec $5\cdot10^{-7}$ M	Urine samples diluted 1:10 with	[39]

	Hg/Hg_2Cl_2, KCl (sat.) ∣ samples solution ∣∣ membrane ∣∣ graphite rod	−2.02; NO_3^- −2.77; ClO_4^- −2.36; NO_2^- −2.44; CN^- −2.34; N_3^- −2.27; F^- −2.85; Cl^- −2.80; Br^- −2.54; Sal^- −2.08; I^- −2.27	10^{-1} – 10^{-6} M	0.005 M phosphate buffer.<br>Potentiometric titration of SCN^- with $AgNO_3$ solution.	
nickel phthalocyanine<br>**(I-2)**	5.1% ionophore, 63% DOP, 31.9% PVC;<br>Hg/Hg_2Cl_2, KCl (sat.) ∣ samples solution ∣∣ membrane ∣∣ graphite rod	**SSM**: CH_3COO^- −4.4; SO_4^{2-} −4.9; $H_2PO_4^-$ −3.6; CO_3^- −2.8; Oxalate −4.4; NO_3^- −2.6; ClO_4^- −0.4; NO_2^- −2.4; CN^- −1.6; N_3^- −2.1; F^- −2.7; Cl^- −2.5; Br^- −2.2; Sal^- −2.6; I^- −1.3	−58.4 mV/dec<br>$5 \cdot 10^{-7}$ M<br>10^{-1} – $5 \cdot 10^{-7}$ M	Urine samples diluted 1 : 10 and adjusted to pH 5.0.	[40]
iron(III) phthalocyanine chloride<br>**(I-2)**	5.2% ionophore, 62.8% DOP, 32% PVC;<br>Hg/Hg_2Cl_2, KCl (sat.) ∣ samples solution ∣∣ membrane ∣∣ graphite rod	**SSM:** CH_3COO^- −2.6; SO_4^{2-} −4.0; $H_2PO_4^-$ −2.6; CO_3^{2-} −2.1; Oxalate −4.1; NO_3^- −2.6; ClO_4^- −0.6; NO_2^- −2.5; CN^- −3.3; N_3^- −2.1; F^- −2.6; Cl^- −2.5; Br^- −2.2; Sal^- −2.7; I^- −1.5	−57.2 mV/dec<br>$2 \cdot 10^{-6}$ M<br>10^{-1} – $5 \cdot 10^{-6}$ M	Urine samples diluted 1 : 10 and adjusted to pH 5.	[40]
Mn(II) complex of N,N'−bis−(4−phenylazosalicylidene) o−phenylene diamine<br>**(I-6)**	3.81% ionophore, 30.44% PVC, 65.45% DOP, 0.3% DTOAI<br>$Hg–Hg_2Cl_2$ ∣ KCl (sat.) ∣ (sample solution ∣∣ membrane∣∣ 0.01 M KCl ∣ AgCl – Ag.	**SSM:** I^- −1.34;Sal – 1.44; Benzoate −2.28; ClO_4^- –1.40; NO_3^- –4.59; NO_2^- –3.12; Br^-–4.05; AcO^- –4.61; Cl^- –4.84	−57.3 mV/dec<br>LOD: nd<br>10^{-1} – $7.02 \cdot 10^{-6}$ M	Waste water samples.	[48]
cadmium {*N*,*N*′−bis(salicylidene)−1,2−ethylenediamine} complex<br>**(I-6)**	30% PVC, 7% ionophore, 3% HTAB (hexadecyl trimethyl ammonium bromide) and 60% DBP (dibutyl phthalate).<br>Ag/AgCl ∣∣ KCl (3 M) internal solution, 1.0×10^{-3} M KSCN ∣ PVC	**MPM**: CH_3COO^- −4.6; SO_4^{2-} −3.3; SO_3^{2-} −3.3; CO_3^{2-} −3.4; NO_3^- −3.1; ClO_4^- −3.0; NO_2^- −3.5; CN^- −3.3; Cl^- −3.0; Br^-−3.2; I^- −2.5; MnO_4^- −1.7; $S_2O_3^{2-}$ −3.3; IO_3^- −3.8; HCO_3^- −3.4; Sal^-	−59.1 mV/dec<br>$7 \cdot 10^{-7}$ M<br>10^{-1} – $5 \cdot 10^{-6}$ M	Determination of thiocyanate in a sample of milk.	[49]

	membrane ∣ test solution ∣ Hg–Hg_2Cl_2, KCl (sat.).	−2.8; Succinate −3.4; Citrate −3.9; Ascorbate −3.6			
1,8−dibenzyl−1,3,6,8,10,13−hexaazacyclotetradecane–Ni(II) Perchlorate<br>**(I-3)**	6% ionophore, 60.2% DBP, 2.7% sodium tetraphenyl borate (NaTPB), 31.1% PVC<br><br>Pt−electrode/PVC membrane/sample solution/KCl(sat.*)*, Hg_2Cl_2/Hg	FIM: NO_2^- −4.0; Saicylate −2.1; Oxalate−1.9; Citrate -3.9; Br^- −1.8; IO_3^-−2.5; SO_4^{2-} −3.1; CH_3COO^- -3.0; HCO_3^- −2.5; NO_3^- −1.8; $S_2O_3^{2-}$ −3.3; I^- −3.2; MnO_4^- −4.2; CrO_4^{2-} −2.7; ClO_4^- −3.2; ClO_3^- −3.5; Cl^- −3.2	−58.4 mV/dec<br>$3{\cdot}10^{-6}$ M<br>$3.3{\cdot}10^{-6}$ – 0.10 M	Urine and saliva samples diluted 1:10 with water and adjusted to pH 5.0 with phosphoric acid and/or potassium hydroxide solution; potentiometric titration.	[42]
chloro[5,10,15,20−tetra−phenyl)porphyrinato]manganese(III)<br>**(I-1)**	28% PVC, 57% DOP, 10% ionophore and 5%NaTPB<br><br>Hg/Hg_2Cl_2, KCl (sat.) ∣ samples solution ∣∣ membrane ∣∣ graphite rod	**SSM**: CH_3COO^- −3.66; SO_4^{2-} −5.35; $H_2PO_4^-$ −4.38; CO_3^{2-} −2.75; Oxalate −5.44; NO_3^- −2.9; ClO_4^- −1.54; NO_2^- −3.88; CN^- −3.71; N_3^- −2.73; F^- −3.44; Cl^- −3.88; Br^- −2.86; Sal^- −1.9; I^- −2.49	−59.5 mV/dec<br>$5{\cdot}10^{-8}$ M<br>10^{-1} – 10^{-7} M	Urine samples diluted 1:10 with 0.05 M phosphate buffer at pH 4.5.	[37]
2,4,10,12−tetramethyl−1,5,9,13−(benzo) tetrazacyclopentadecinato (2−) nickel(II)<br>**(I-3)**	5.7% ionophore, 62.7% NPOE, 31.6% PVC<br><br>Ag, AgCl∣1–2 drop of KCl (sat.), $1.0{\times}10^{-3}$ M KSCN∣PVC membrane∣test solution∣Ag, AgCl, KCl (sat.).	**FIM**: CH_3COO^- −3.08; N_3^- −2.45; SO_4^{2-} −4.20; PO_4^{3-} −3.40; CO_3^{2-} −3.15; Oxalate −3.00; NO_3^- −2.2; ClO_4^-−0.95; −2.7; CN^- −1.65; F^- −2.30; Cl^- −3.10; Br^- −2.45; Sal^- −2.2; I^- −1.10; S^{2-} − 2.91; $S_2O_3^{2-}$ −3.55; Formate −2.53, Citrate −3.34; NO_2^- −2.2; Phthalate −2.3; Succinate −3.7; Tartarate −3.75	−59.7 mV<br>$1.4{\cdot}10^{-7}$ M<br>10^{-1} – $1.4{\cdot}10^{-7}$ M	Urine samples diluted 1:10 (or 1:20) and adjusted to pH 5.0 with H_3PO_4 or KOH solutions.<br><br>Saliva− no info about pretreatment, potentiometric titration.	[43]
Ni(II) 2,2,4,9,9,11−hexamethyltetraazacyclotetradekanediene perchlorate<br>**(I-3)**	5% ionophore, 63% DBP, 2% HTAB, 30% PVC	**MPM:** F^- −4.49; Cl^- −4.37; Br^- −4.15; I^- −2.64; $S_2O_3^{2-}$	−57.8 mV/dec	Bovine milk, urine or saliva diluted 1:10	[44]

	Hg/Hg_2Cl_2, KCl (sat.) ∣ samples solution ∣∣ membrane ∣∣ graphite rod	−3.23; SO_3^{2-} −4.30; HCO_3^- −4.05; CO_3^{2-} −4.18; NO_2^- −3.51; CN^- −2.72; ClO_4^- −3.15; ClO_3^- −3.09; IO_3^- −3.07; IO_4^- −3.14; CrO_4^{2-} −4.35; $Cr_2O_7^{2-}$ −−4.42; MnO_4^- −4.54; MoO_4^{3-} −4.68	$4.8·10^{-8}$ M 10^{-1} – 10^{-7} M	and adjusted to pH 5.0 with H_3PO_4 or KOH solutions.	
		**SSM:** F^- −4.51; Cl^- −4.48; Br^- −4.31; I^- −2.48; $S_2O_3^{2-}$ −4.32; SO_3^{2-} −3.16; HCO_3^- −4.11; CO_3^{2-} −3.09; NO_2^- −3.15; CN^- −2.57; ClO_4^- −3.18; ClO_3^- −3.17; IO_3^- −3.10; IO_4^- −3.15; CrO_4^{2-} −4.15; $Cr_2O_7^{2-}$ −4.15; MnO_4^- −4.75; MoO_4^{3-} −4.96			
bis−bebzoin−semitriethylenetetraamine binuclear copper(II) **(I-6)**	6.5 % ionophore, 64.9% o−NPOE, 28.6% PVC Hg/Hg_2Cl_2, KCl (sat.) ∣ samples solution ∣∣ membrane ∣∣ 0.1 M KCl Ag/AgCl	**SSM:** Cl^- −2.7; I^- 0.9; Sal −1.0; ClO_4^- −0.5; NO_3^- −2.5; NO_2^- −2.8; SO_4^{2-} −3.4; SO_3^{2-} −2.0; $H_2PO_4^-$ −2.6	−57.0 mV/dec $7·10^{-7}$ M 10^{-1} – $9·10^{-7}$ M	Industrial wastewater sample.	[50]
Ionophore II (I-6)	1.5% ionophore, 65% DOP, 33 % PVC, 0.5% TDMAC Ag/AgCl(*s*), 4 M KCl (sat.). with AgCl(s) ∣ sample solution ∣∣ membrane ∣∣ internal reference solution/AgCl(*s*)/Ag.	**SSM**: no given values, diagram only IO_4^-> SCN^->I^-> ClO_3^-> HCO_3^-> CN^- > NO_3^- > Br^- ~CH_3COO^-> NO_2^-> BrO_3^- > F^-> CrO_4^->S^{2-} > $S_2O_3^{2-}$> $H_2PO_4^-$ > SO_4^{2-} > SO_3^{2-}	−56.3 mV/dec $5.5·10^{-6}$ M 10^{-5} – $1.0·10^{-2}$ M	Potentiometric titration of Ag^+, Cu^{2+}, Tl^+ and Pb^{2+}.	[51]
bis(2−mercaptobenzoxazolato) mercury(II) **(I-6)**	30.5% PVC, 61.0% DOP, 6% ionophore, 2.5% methyltrioctylammonium	**FIM:** CH_3COO^- −4.12; N_3^- −3.40; SO_4^{2-} −4.35; PO_4^{3-} −4.10; CO_3^{2-} −3.92; Oxalate	−60.6 mV/dec $6·10^{-7}$ M	Saliva diluted 1: 10 with 0.05 M	[52]

	chloride<br>Ag, AgCl ∣ KCl (sat.) ∣ membrane∣∣ test solution∣ graphite electrode	−4.62; NO_3^- −4.82; ClO_4^- −1.46; NO_2^- −3.36; CN^-; F^- −3.82; Cl^- −0.99; Br^- −2.12; Sal^- −3.44; I^- −0.65; CO_3^{2-} −3.92	10^{-1} – 10^{-6} M	phosphate buffer of pH 5.<br>Potentiometric titration of SCN^- with $AgNO_3$ solution.	
two zinc(II) ions and two molecules of the bis−N,O−bidentate Schiff base 2,2_−[methylenebis(4,1−phenylenenitrilomethylidyne)]bisphenol **(I-6)**	5% ionophore, chloroparaffin−PVC (2 :1), Tridodecylmethylammonium chloride (TDDMACl)<br>Ag∣ AgCl ∣3 M KCl ∣∣ 1 M $LiOCOCH_3$ ∣∣sample∣ membrane ∣ internal filling solution ∣AgCl∣ Ag	**FIM:** CH_3COO^- −3.6; N_3^- −2.1; SO_4^{2-} −2.0; $H_2PO_4^-$ −3.8; HPO_4^{2-} −2.7; HCO_3^- −3.3; NO_3^- −1.5; ClO_4^- −0.3; NO_2^- −2.3; CN^- −2.6; F^- −4.0; Cl^- −2.7; Br^- −2.1; Sal^- −0.3; I^- −0.5;	−57.5 mV/dec<br>$4{\cdot}10^{-7}$ M<br>LR: nd	No sample.	[53]
N,N'−bis−(benzaldehyde)−glycine copper(II) complex **(I-6)**	3.0% ionophore, 67.2% *o*−NPOE and 29.8% PVC<br>Hg−Hg_2Cl_2/KCl (sat.)/sample solution/membrane/ 0.1 M HCl/AgC1−Ag	**FIM: :** Cl^- −3.9; I^- −2.0; Sal −1.8; ClO_4^- −1.6; NO_3^- −3.5; NO_2^- −3.0; SO_3^{2-} −3.1; $H_2PO_4^-$ −4.4; Br^- −2.4<br>**SSM:** Cl^- −4.3; I^- −2.4; Sal −2.1; ClO_4^- −1.8; NO_3^- −3.7; NO_2^- −3.3; SO_3^{2-} −4.1; $H_2PO_4^-$ −4.5; Br^- −2.7	−57.6 mV/dec<br>$7{\cdot}10^{-7}$ M<br>10^{-1} – $9{\cdot}10^{-6}$ M	Industrial wastewater sample.<br>Saliva samples adjusted to pH 4.0 with phosphoric acid and/or sodium hydroxide solution.	[54]
Rh[(trpy)(bpy)Cl](PF6)2 **(I-6)**	3% ionophore, 65% o−NPOE and 30% PVC, 2% HDTMAB<br>Ag−AgCl ∣KCl(3 M)∣ internal solution, 1 mM SCN^- ∣PVC membrane∣ test solution Hg−Hg_2Cl_2, KCl(sat.)	**MPM:** F^- −4.7; Cl^- − 4.0; Br^- −3.4; NO_2^- −4.8; NO_3^- −5.0; ClO_4^- −2.2; IO_3^- −3.3; SO_3^{2-} −4.7; SO_4^{2-} −4.8; $S_2O_3^{2-}$ −4.2	−58.7 mV/dec<br>$4{\cdot}10^{-6}$ M<br>10^{-1} – 10^{-5} M	Saliva samples diluted 1:10 with water and adjusted to pH 5.0 with phosphoric acid and/or potassium hydroxide solution.	[55]
butane−2,3−dione bis(salicylhydrazonato)zinc(II) complex **(I-6)**	32% PVC, 60% DOP, 6% ionophore, 2% TOMAC.	**FIM:** Cl^- −3.9; I^- −3.8; Sal −3.2; ClO_4^- −2.8; NO_3^- −3.0; NO_2^- −3.1; SO_3^{2-} −3.1; Br^- −3.3; CN^- −3.5; F^- −4.1;	−56.5 mV/dec<br>$7{\cdot}10^{-7}$ M	Industrial wastewater sample.	[56]

	Ag ∣ AgCl ∣ KCl (3 M) internal solution (10^{-5} M KSCN)∣ PVC membrane ∣ test solution ∣∣ SCE	CO_3^{2-} −4.0; SO_4^{2-} −4.1; HPO_4^{2-} −4.0; PO_4^{3-} −4.2; ClO_3^- −3.2; CrO_4^{2-} −3.4; MnO_4^- −3.4	10^{-1} – 10^{-6} M	Urine and saliva samples diluted 1:10 with water and adjusted to pH 5.0 with phosphoric acid and/or potassium hydroxide solution.	
5,11,17,23−tetra−*tert*−butyl−25,26,27,28−tetracyanometoxy−calix[4]arene **(I-5)**	1% ionophore, 66% 2−NPOE and 33% PVC<br><br>double junction reference electrode/analyte solution/ membrane/1.0×10^{-2} M HCl/Ag/AgCl	**SSM:** I^- 2.882; ClO_4^- 0.593; $H_2PO_4^-$−2.201; AcO^- −2.292; F^- −2.027; NO_3^- −2.347; NO_2^- −2.237; HSO_3^- −2.167; Br^- −1.967	−52 mV/dec<br>2.5·10^{-5} M<br>10^{-1} – 3·10^{-5} M	Potentiometric titration of SCN^- with $AgNO_3$ solution.	[46]
nickel(II)−1,4,8,11,15,18,22,25−octabutoxyphthalocyanine **(I-2)**	30% PVC, 65% DBP, 3% ionophore and 2% hexadecyltrimethylammonium bromide<br><br>Ag/AgCl ∣ internal solution (1 mM KSCN) ∣ PVC membrane ∣ test solution ∣ Ag/AgCl ∣ KCl (sat.).	**MPM:** ClO_4^- −3.04; ClO_3^- −3.15; IO_4^- −4.34; IO_3^- −4.15; CN^- −3.42; NO_3^- −3.50; NO_2^- −4.00; F^- −4.00; Cl^- −4.09; Br^- − 3.70; I^- − 3.4; AcO^- −4.45; SO_4^{2-} −4.02; CO_3^{2-} −4.04	−58.7 mV/dec<br>5.7·10^{-7} M<br>10^{-1} – 10^{-6} M	Potentiometric titration of SCN^- with $AgNO_3$ solution.<br><br>Urine and saliva samples diluted 1:10 with water and adjusted to pH 5.0 with phosphoric acid and/or potassium hydroxide solution.	[41]
12−crown−4−ether−cetyltrimethyl ammonium thiocyanate ion−pair **(I-4)**	5% ionophore, 31% PVC, 64% DOP<br><br>Ag/AgCl(s), 4 M KCl (sat.) with AgCl(s)/sample solution//membrane//graphite	**SSM:** IO_4^- −1.9; Cl^- −4.3; Br^- −4.2; I^- −4.1; SO_4^{2-} −3.9; SO_3^{2-} −3.7; CN^- −4.2; NO_2^- −4.1; NO_3^- −3.7; CrO_4^{2-} −4.6; ClO_4^- −3.7; MnO_4^- −4.5; PO_4^{3-} −4.6; CO_3^{2-} −3.9; BrO_3^- −3.1; AcO^- −3.8; $C_2O_4^{2-}$ −3.7; Sal −3.6	−57.6 mV/dec<br>0.03 μg·ml^{-1}<br>10^{-1} – 10^{-6} M	Monitoring sequential titration of some metal ions (e.g. Ag^+, Cu^+, Pb^{2+}).	[45]

zinc−tris(*N*−*tert*−butyl−2−thioimidazolyl)hydroborate complex **(I-6)**	5% ionophore, 33% PVC, 33% DOP, 2% HTAB<br><br>Ag/AgCl/KCl (sat.)/test solution/PVC membrane/10^{-2} M NaSCN/Hg/Hg_2Cl_2	**MPM:** ClO_4^- −4.2; CO_3^{2-} −4.7; Cl^- −4.4; MnO_4^- −4.0; Br^- −4.3; NO_3^- −2.8; I^- −3.6; SO_4^{2-} −4.3; NO_2^- −4.2; $S_2O_3^{2-}$ −3.4; CN^- −3.5; PO_4^{3-} −4.8; OAc^- −4.9; Sal −3.6; $H_2PO_4^-$ −4.6; $Cr_2O_7^{2-}$ −4.1; $Cr_2O_4^{2-}$ −4.8; F^- −4.2; ClO_3^- −3.8	−59.4 mV/dec<br>$3.16 \cdot 10^{-7}$ M<br>10^{-2} – $6.3 \cdot 10^{-7}$ M	Urine, saliva, and serum samples treated by MES/NaOH buffer (pH 5.5) solution.<br><br>River water samples.	[57]
Ni(II) complex of para−tolualdehydesemicarbazone **(I-6)**	4% ionophore, 31% PVC, 63% TBP, 2% HTAB<br><br>Hg−Hg_2Cl_2 KCl (sat.) ǀǀ internal solution 0.1 mM KI ǀ PVC membrane ǀ test solution ǀ Hg_2Cl_2−Hg, KCl (sat.)	**MPM:** I^- −3.8; NO_3^- −4.3; Cl^- −4.0; HCO_3^- −4.7; SO_4^{2-} −4.6; Br^- −4.3; CrO_4^- −3.0; F^- −4.1; CO_3^{2-} −3.8; $S_2O_6^{2-}$ −3.2; $S_2O_3^{2-}$ −3.6	−58.8 mV/dec<br>$1.25 \cdot 10^{-7}$ M<br>10^{-2} – 10^{-7} M	Urine samples diluted 1:10 with water and adjusted to pH 4.5 with phosphate buffer.<br><br>Potentiometric titration of SCN^- with $AgNO_3$ solution.	[58]
[hydrotris(3−phenyl−5−methyl−1−pyrazolyl)borate](3−phenyl−5−methyl−pyrazole) nickel chloride **(I-6)**	7% ionophore, 2% HTAB, 60% DBP, 31% PVC<br><br>Hg,Hg_2Cl_2 ǀKCl(satd.)ǀ0.01M ǀǀPVCmembraneǀǀ test solutionǀ Hg,Hg_2Cl_2ǀKCl(sat.)	**MPM:** ClO_4^- −2.27; N_3^- −6.21; Cl^- −4.64; Br^- −4.44; CN^- −1.15; NO_2^- −3.92; NO_3^- −3.72; AcO^- −3.08; $H_2PO_4^-$ −2.48; CO_3^{2-} −3.24; SO_4^{2-} −4.61; OH^- −3.5	−58.8 mV/dec<br>$1.8 \cdot 10^{-7}$ M<br>10^{-2} – $5.3 \cdot 10^{-7}$ M	Urine and saliva samples treated by MES/NaOH buffer (pH 5.5) solution.<br><br>River water samples, pH adjusted to 5.5 with HCl.	[59]
Cobalt (Salpen) (PBu_3)] $ClO_4 \cdot$H2O (Salpen = bis(salycil aldehyde)propylene diamine) **(I-6)**	2% ionophore, 2% MTOAC, 65.5% DOP, 30.7% PVC<br><br>Hg/Hg_2Cl_2/KCl (sat.) ǀǀ test solution ǀ PVC membrane ǀ graphite electrode	**SSM:** ClO_4^- −0.89; Cl^- −3.90; Br^- −3.65; F^- −4.00; I^- −1.21; N_3^- −3.47; CN^- −3.40; NO_2^- −2.33; AcO^- −3.70; $C_2O_4^{2-}$ −2.98; ClO_3^- −1.98; NO_3^- −2.70; CrO_4^{2-} −3.37; IO_3^- −3.62; Imidazol −4.28	−59.05 mV/dec<br>$8 \cdot 10^{-7}$ M<br>10^{-1} –10^{-6} M	Urine and saliva samples diluted 1:10 with water and adjusted to pH 5.2 with HNO_3 or KOH solution.	[60]

manganese complex of N,N′−bis−(4−phenylazosalicylidene)-o-phenylene diamine **(I-6)**	4% ionophore, 66% DBP, 30% PVC<br><br>Pt/electro−conductive polymer/PVC film with an ionophore	**MPM:** ClO_4^- −0.81; Cl^- −3.82; Br^- −3.59; F^- −3.96; I^- −1.16; N_3^- −3.39; CN^- −3.36; NO_2^- −2.30; AcO^- −3.67; $C_2O_4^{2-}$ −2.92; ClO_3^- −1.90; NO_3^- −2.66; CrO_4^{2-} −3.23; IO_3^- −3.56; Imidazol −4.20<br><br>**SSM:** $SCN^- > CrO_4^{2-} > I^- > ClO_4^- > Cr_2O_7^{2-} > ClO_3^- > S^{2-} > SO_3^{2-} > Br^- > NO_3^- > NO_2^- > S_2O_3^{2-} > SO_4^{2-} = AcO^- > IO_3^- > Cl^- > CN^-$	−58.1 mV<br>LOD: nd<br>10^{-1} –$10^{-5.8}$ M	Laboratory wastewater samples (pH adjusted to 5.2 with HNO_3).<br><br>Potentiometric titration of SCN^- with $AgNO_3$ solution.<br><br>Artificial serum.<br><br>Potentiometric titration of SCN^- with $AgNO_3$ solution.	[61]
bis(N−3−methylphenyl salicylidenaminato)copper(II) **(I-6)**	3% ionophore, 31% PVC, 63% DBP, 3% HTAB<br><br>Ag, AgCl, KCl (3 M) ∣ internal solution, KSCN (1mM) ∣ membrane ∣ test solution ∣ KCl (3 M) AgCl, Ag	**FIM:** CN^- −3.0; Cl^- −3.3; CO_3^{2-} −3.8; SO_4^{2-} −3.7	−59.3 mV/dec<br>$5.0 \cdot 10^{-7}$ M<br>10^{-1} – 10^{-6} M	Urine and saliva samples diluted 1:10 with water and adjusted to pH 5.0 with phosphoric acid and/or potassium hydroxide solution.	[62]
Cu(II) sulfadimidine complexes (CuSD) **(I-6)**	7%CuSD; 2.5% CPC; 53% 2−NPOE; 37.5% C<br><br>Ag//AgCl, KCl(3M) // test solution// filling graphite modified paste//carbon paste electrode	**MPM:** Cl^- −3.06; Br^- −2.51; NO_3^- −2.38; NO_2^- −2.91; SO_4^{2-} −2.69; I^- −0.44; F^- −3.77; SO_3^{2-} −-3.06; ClO_4^- −0.36; CO_3^{2-} −2.00; HCO_3^- −2.20 CH_3COO^- −2.00<br><br>**SSM:** Cl^- −1.36; Br^- −1.06; NO_3^- −1.52; NO_2^- −2.01;	−60.6 mV/dec<br>$6.9 \cdot 10^{-7}$ M<br>$1.17 \cdot 10^{-2}$ – $1.82 \cdot 10^{-6}$ M	Thiocyanate content in waste water collected from granite factory.<br><br>Potentiometric titration of SCN^- with $AgNO_3$ solution;	[63]

5,10,15,20-tetraphenylporphine iron(III) chloride **(I-1)**	1% ionophore, 65.5% *o*-NPOE, 32.7% PVC, 0.8% CTAB<br><br>Electronic tongue:<br>5 membrane-coated graphite electrodes in array	SO_4^{2-} −4.71; I^- −0.71; F^- −1.86; SO_3^{2-} −4.62; ClO_4^- −0.63; CO_3^{2-} −0.22; HCO_3^- +0.26; CH_3COO^- +0.27<br>**Measured in the array**<br>**FIM:** Cl^- −3.64; Br^- −3.55; NO_3^- <−5.00; SO_4^{2-} <−5.00; I^- −3.11; F^- −4.23; S^{2-} −3.22; CO_3^{2-} −3.13; HPO_4^{2-} −4.13; CH_3COO^- −3.92; N_3^- −3.25; CN^- −1.73	No data	Urine samples diluted 1:10 and adjusted to pH 5.59.<br><br>Synthetic solution with and without the presence of interfering ions.	[38]
Bis nitrato [4−hydroxyacetophenonesemicarbazone) nickel(II) complex **(I-6)**	31% PVC, 63% 2−NPOE, 4.0% ionophore and 2.0% trioctylmethyl ammonium chloride<br><br>Hg/Hg_2Cl_2(s) ∣ KCl(sat.)∣internal solution 0.1 mM KSCN ∣ ∣ PVC membrane ∣ test solution∣ Hg/Hg_2Cl_2(s) ∣ KCl(sat.)∣	**MPM:** SO_4^{2-} −1.9; NO_3^- −2.8; $Cr_2O_7^{2-}$ −4.4; PO_4^{3-} −3.6; CO_3^{2-} −4.2; Br^- −3.8; $C_2O_4^{2-}$ −2.6; AcO^- −2.9; Cl^- −2.4; I^- −1.1; NO_2^- −2.1	−59.4 mV/dec<br>$8.6 \cdot 10^{-8}$ M<br>10^{-1} – 10^{-7} M	Urine samples diluted 1:10 with water and adjusted to pH 5.0 with phosphoric acid and/or potassium hydroxide solution.<br><br>Titration.<br><br>Determination of SCN^- in river and tap water.	[64]

Table S2. Comparison of mean concentrations of SCN^- in the relation of answers given in the survey.

**Parameter**		**N**	**SCN^- (mM)**	
			$\bar{x}$	**SD**
Sex	Women	68	0.85	0.58
	Men	32	0.85	0.56
Drugs	Yes	69	0.80	0.52
	No	31	0.97	0.67
	Supplements	35	0.78	0.53
	Antibiotics	7	0.74	0.45
	OTC	38	0.83	0.55
	Steroids	6	0.87	0.45
	Hormonal drugs	5	0.66	0.35
	Contraceptive	11	0.88	0.59
	Other	15	0.78	0.54
Fluids	Water	43	0.84	0.57
	Tea	29	0.81	0.57
	Coffee	16	0.78	0.51
	Juice	9	1.17	0.59
	Drink	3	1.00	0.28
	1 L	12	1.09	0.72
	2 L	40	0.82	0.50
	2.5 L	40	0.82	0.59
	More than 3 L	8	0.90	0.24
Contraceptive	Yes	13	0.87	0.59
	No	55	0.86	0.59
Chronical diseases	Yes	30	0.79	0.51
	No	70	0.87	0.59
	Renal	1	1.02	---
	Cardiovascular	3	0.86	0.53
	Hematological	3	0.88	0.26
	Pulmonary	6	0.69	0.54
	Allergies	7	0.70	0.54
	Other	10	0.88	0.41
Brushing teeth	Today morning	95	0.86	0.58
	Yesterday evening	4	0.58	0.42
	Earlier than yesterday	1	0.85	---
Frame of mind	Good	32	0.91	0.64
	Fatigue	53	0.82	0.49
	Infection	4	0.68	0.43
	Chronic desease	11	0.75	0.55
Oral hygiene liquid	Yes	27	0.83	0.65
	No	73	0.85	0.55

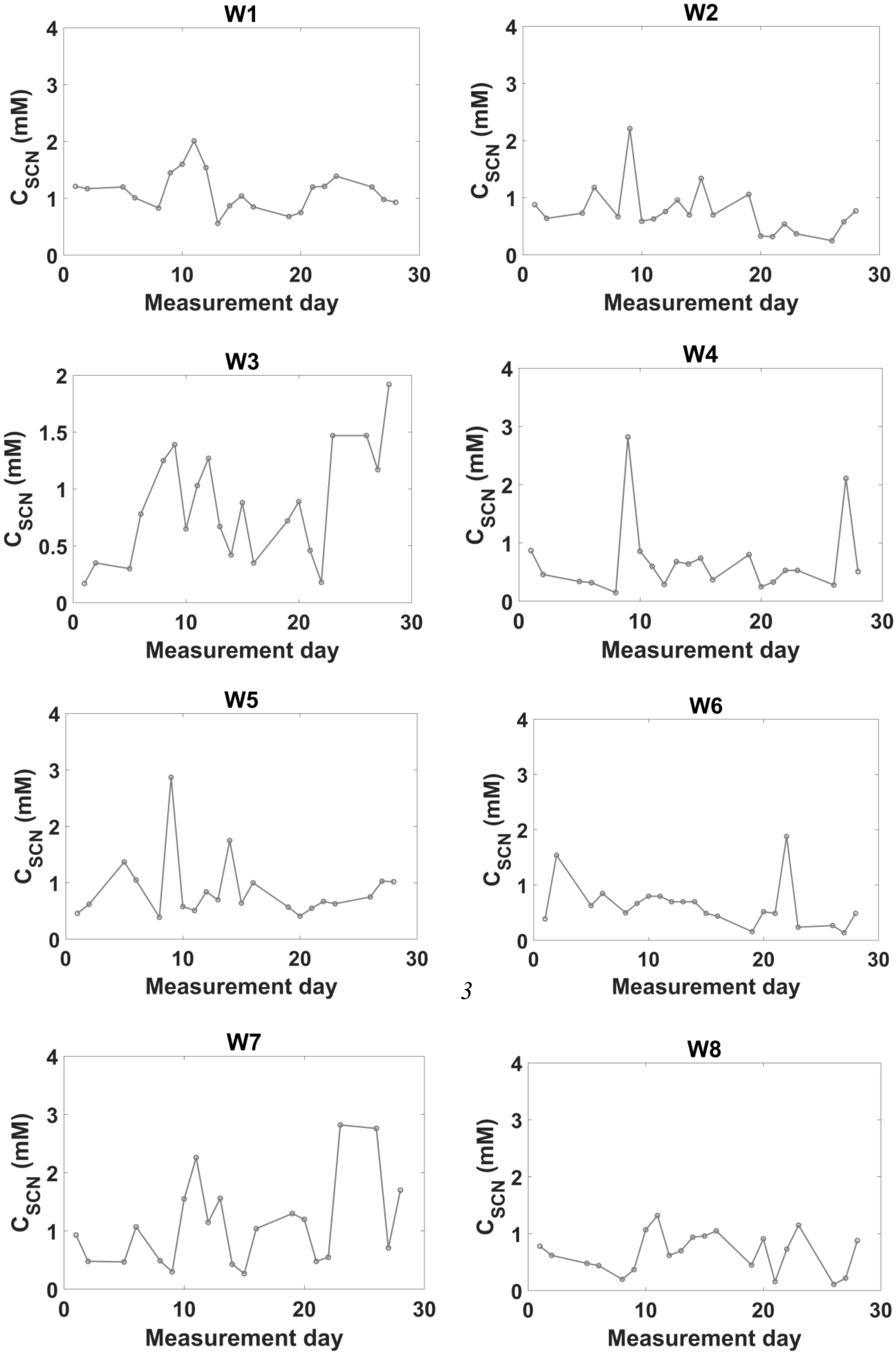

3

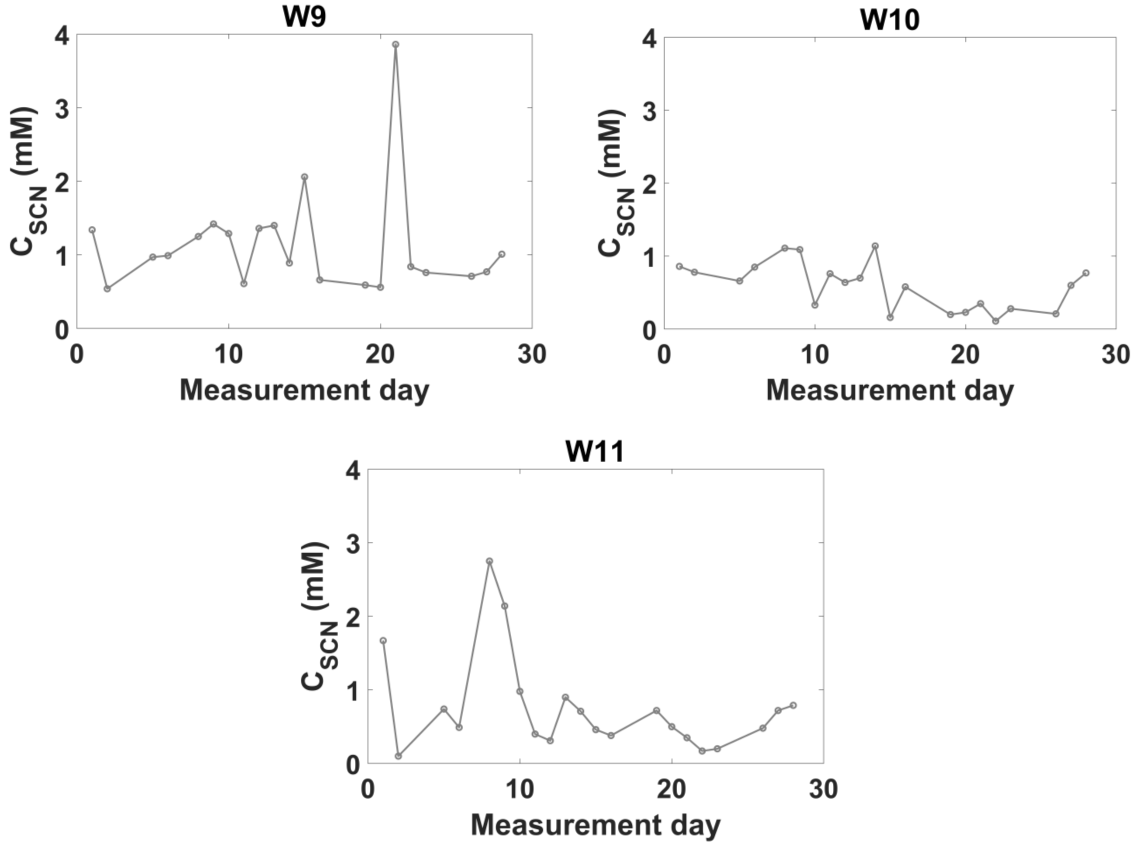

Figure S1. Monthly ionograms showing fluctuations of SCN- concentration for selected women.

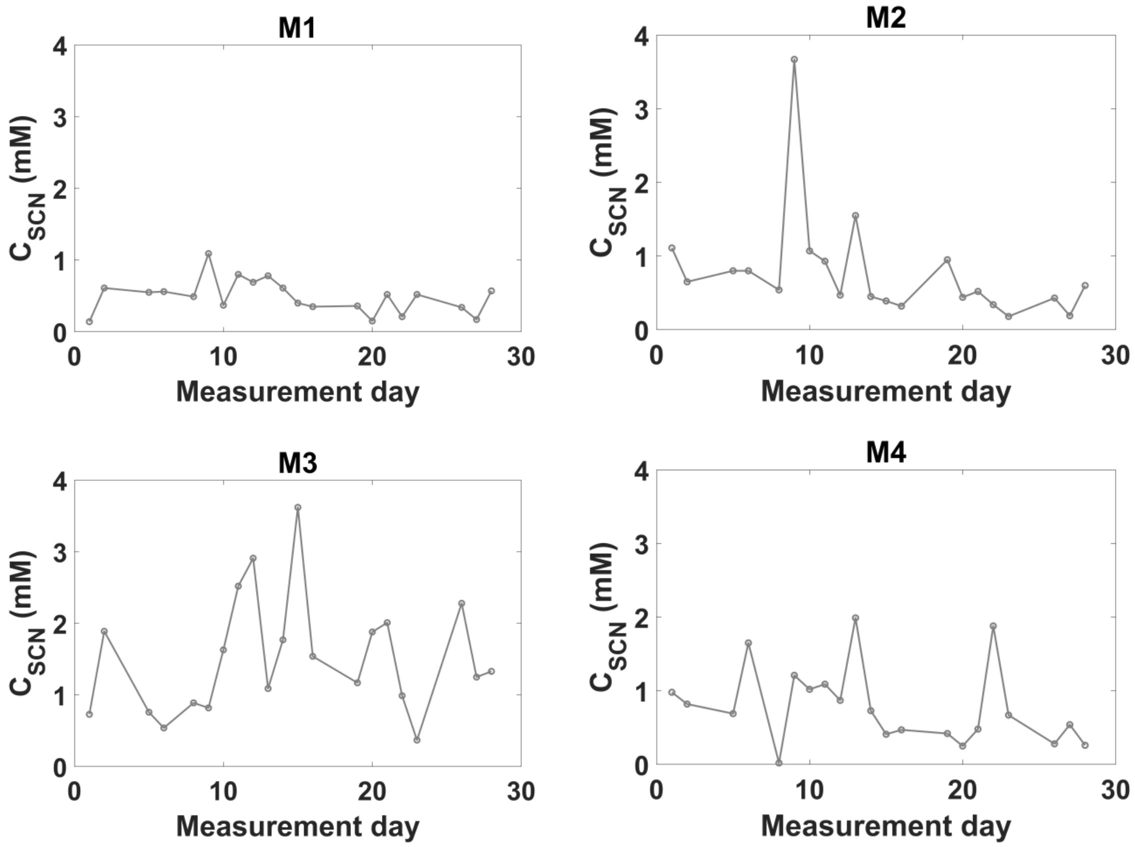

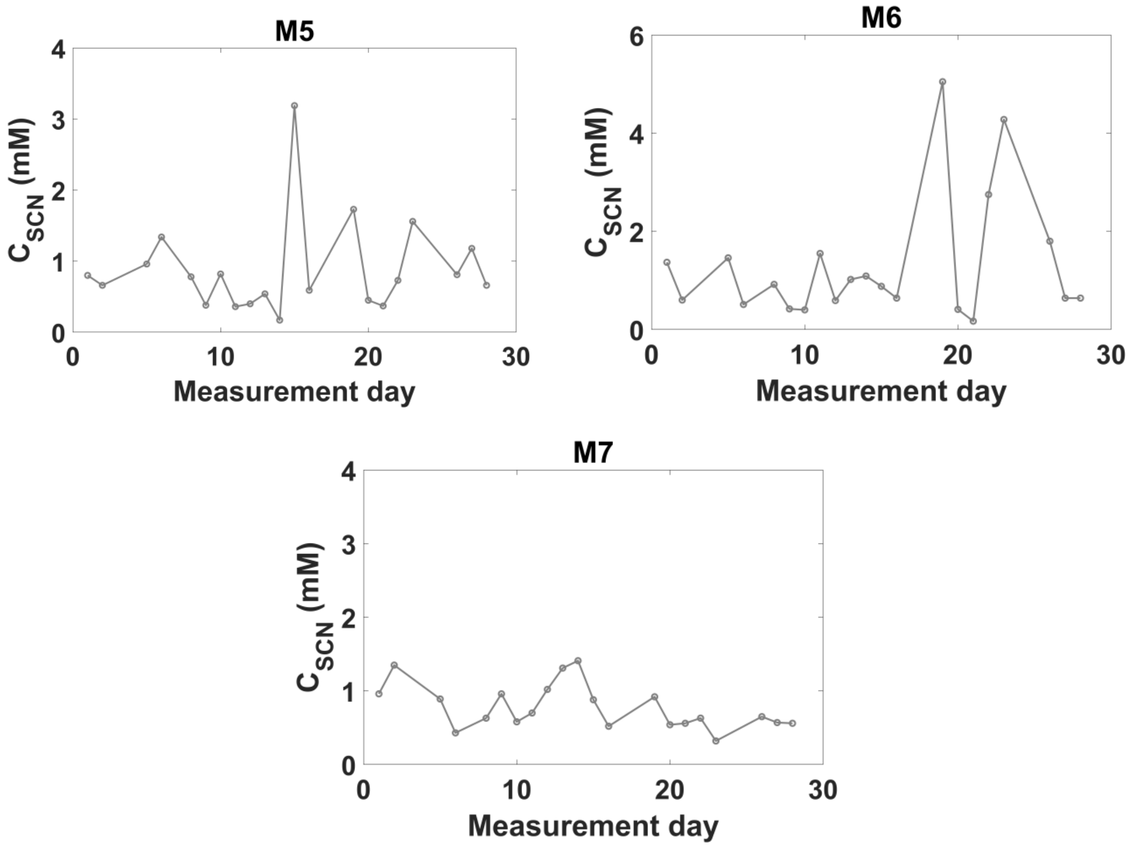

Figure S2. Monthly ionograms showing fluctuations of SCN- concentration for selected men.
